# HDL as a drug and nucleic acid delivery vehicle

**DOI:** 10.3389/fphar.2015.00247

**Published:** 2015-10-26

**Authors:** Andras G. Lacko, Nirupama A. Sabnis, Bhavani Nagarajan, Walter J. McConathy

**Affiliations:** ^1^Department of Integrative Physiology and Anatomy, University of North Texas Health Science Center, Fort Worth, TX, USA; ^2^Department of Pediatrics, University of North Texas Health Science Center, Fort Worth, TX, USA; ^3^Lipomedics Inc., Fort Worth, TX, USA

**Keywords:** lipoproteins, HDL, drug delivery, nucleic acid delivery, therapeutics

## Abstract

This review is intended to evaluate the research findings and potential clinical applications of drug transport systems, developed based on the concepts of the structure/function and physiological role(s) of high density lipoprotein type nanoparticles. These macromolecules provide targeted transport of cholesteryl esters (a highly lipophilic payload) in their natural/physiological environment. The ability to accommodate highly water insoluble constituents in their core regions enables High density lipoproteins (HDL) type nanoparticles to effectively transport hydrophobic drugs subsequent to systemic administration. Even though the application of reconstituted HDL in the treatment of a number of diseases is reviewed, the primary focus is on the application of HDL type drug delivery agents in cancer chemotherapy. The use of both native and synthetic HDL as drug delivery agents is compared to evaluate their respective potentials for commercial and clinical development. The current status and future perspectives for HDL type nanoparticles are discussed, including current obstacles and future applications in therapeutics.

## HDL as a Transport Vehicle

**FIGURE 1:**
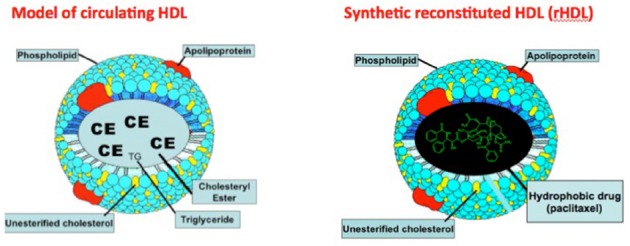
**Localization of a hydrophobic drug (paclitaxel) in the core region of synthetic (reconstituted) HDL (rHDL).** Adapted from Weinberg (1987).

High density lipoproteins (HDL) participate in reverse cholesterol transport in mammals to deliver the peripherally accumulated excess cholesterol to the liver (Rader et al., 2009). The structure of HDL is superbly suited for the transport of cholesteryl esters and lipophilic compounds, including drugs (Figure 1) in its core compartment so that the cholesterol payload can safely reach its destination (Lund-Katz and Phillips, 2010). The same advantages apply to the transport of other lipophilic payloads, including drugs that can be delivered to specific cell/tissue targets via HDL type carriers (Lacko et al., 2002, 2007; Sabnis and Lacko, 2012). Despite the growing interest in HDL as a drug delivery/therapeutic agent (Ng et al., 2011; Huang et al., 2015; McMahon et al., 2015), clinical studies have yet to be initiated with this transport vehicle. While recent literature reports in this area suggest a marked upsurge in research activity that rely on reconstituted HDL (rHDL) type nanoparticles, major review articles and books often ignore lipoprotein based drug delivery, in favor of sophisticatedly engineered and often more complex and potentially much more expensive nanoparticles. For example, a recent multi-volume treatise on “Nanobiomedical Research” contains no information on lipoprotein based drug delivery whatsoever. Perhaps a breakthrough finding regarding enhanced therapeutic potential of drugs will provide the momentum for the development of HDL type therapeutic transport vehicles.

## HDL as a Drug Delivery Vehicle

The use of lipoproteins and particularly HDL (Schouten et al., 1993) as drug delivery agents was first suggested over 30 years ago (Counsell and Pohland, 1982). Subsequently, Kader and Pater (2002) evaluated HDL particles as drug transporters. They have found that drugs incorporated into HDL were 2.5–23 times cytotoxic to cancer cells than the free (non-encapsulated) drug. Despite these promising findings, the use of plasma lipoproteins as drug carriers has been limited by the lack of their consistent composition (depending on the source) and the potential for harboring infectious agents. Since then significant research activity followed in this area using synthetic/rHDL nanoparticles (Lacko et al., 2007; Ng et al., 2011; Huang et al., 2015; McMahon et al., 2015). The slow development of HDL type drug formulations toward clinical applications is surprising as these nanoparticles have numerous advantageous characteristics, including:

(1) Enhanced safety and efficacy yielding a sturdy, non-leaking preparation due to a structure stabilized by apolipoproteins, particularly apolipoprotein A-I (apo A-I).
(2) Biocompatibility and safety, shown by several pharmaceutical formulations, made up of essentially the same ingredients as rHDL (Kujiraoka et al., 2003; Hovingh et al., 2015; Kootte et al., 2015).
(3) The receptor-mediated uptake of the payload, carried in the core of the HDL particle (Figure 1) makes this strategy uniquely applicable, especially for cancer chemotherapy (Lacko et al., 2007; Ng et al., 2011).

## Synthetic/Reconstituted HDL as a Drug Delivery Vehicle

Because of the concerns about infectious agents and the reproducibility of samples of human plasma HDL, its use for drug delivery in a clinical setting seems impractical. Consequently, most of the research activity in the last 20 years has focused on the development and evaluation of rHDL drug delivery vehicles (Table 1). In a previous review (Lacko et al., 2007), we discussed the physical chemical characteristics of the rHDL drug delivery particles, especially regarding the discrepancy in the preparatory procedures and the shape and size of the resultant particles. Since then, there has been an effort to identify discoidal particles as “nanodisks” (Tufteland et al., 2008) or discoidal particles to differentiate them from spherical structures (Wang et al., 2013). The latter rHDL particles have been assembled using a variety of methods. Including sonication (Zhang et al., 2009) and detergent dialysis (McConathy et al., 2008) producing largely spherical, HDL like nanoparticles. The distinction between the discoidal and spherical rHDL particles is likely to be physiologically and pharmacologically significant as their respective drug carrying capacities and metabolism are likely to be different.

**TABLE 1:** **Landmarks in the development of HDL type nanoparticles for the delivery of drugs and nucleic acids**.

**Findings and conclusions**	**Reference**
Proposed lipoproteins as vehicles for site-specific delivery of radiopharmaceuticals.	Counsell and Pohland (1982)
Lactosylated apo E containing (synthetic) neo-HDL is rapidly taken up by parenchymal liver cells.	Schouten et al. (1993)
Loading anticancer drugs into HDL or LDL enhances their cytotoxicity toward cancer cells.	Kader and Pater (2002)
Reconstituted HDL can effectively deliver drugs to cancer cells due to their over expression of the SR-B1 receptor.	Lacko et al. (2002)
rHDL-paclitaxel nanoparticles had superior cytotoxicity against prostate, breast and ovarian cancer cells (compared to the free drug) and were better tolerated by mice than either Taxol or Abraxane.	McConathy et al. (2008)
rHDL nanoparticles utilized for cytosolic delivery of drugs.	Zhang et al. (2009)
Assembly of gold containing rHDL nanoparticles for drug delivery.	Thaxton et al. (2009)
rHDL nanoparticles containing apo A-I mimetic peptide for drug delivery to malignant tumors.	Zhang et al. (2010)
The SR-B1 receptor is primarily (82%) responsible of for the uptake of paclitaxel from rHDL nanoparticles by prostate cancer (PC-3) cells.	Mooberry et al. (2010)
rHDL delivered STAT3 siRNA contributed to 90% suppression of ovarian tumor xenografts while inducing a fivefold increase in apoptosis and a threefold decrease in tumor angiogenesis. Tumor uptake of the siRNA form rHDL appeared to be facilitated by the SR-B1 receptor.	Shahzad et al. (2011)
Improved therapeutic efficacy of anti-cancer agents via encapsulation into rHDL nanoparticles.	Sabnis et al. (2012, 2013)
rHDL particles can be prepared via a microfluidizer.	Kim et al. (2013)
Super paramagnetic iron oxide can be encapsulated into rHDL nanoparticles along with drugs and directed toward their target in a magnetic field.	Sabnis et al. (2015)

## Apo A-I Mimetic Peptides as Components of HDL Type Drug Delivery Vehicles

Commercial development of HDL drug delivery vehicles has been hampered, at least to a degree, by the availability of the protein component apo A-I (Ryan et al., 2003). Sufficient quantities of human apo A-I for scaling up and eventual industrial production are currently not commercially available.

Alternatives to apo A-I or other full length apolipoproteins may be appropriately found via the application of mimetic peptides (Remaley et al., 2003) in the assembly of rHDL nanoparticles (Zhang et al., 2010). These peptides are designed to contain amphipathic helical structures, similar to the helical bundles that have been identified in apo A-I (Ditiatkovski et al., 2013; Leman et al., 2014). The apo A-I mimetic peptides thus, for the most part, mimic the function of apo A-I by contributing to lipoprotein assembly (Zhang et al., 2010) and receptor interactions. Interestingly, the apolipoprotein component of HDL/rHDL (apo A-I) has been found to have anti-tumor effect on its own (Zamanian-Daryoush et al., 2013), perhaps due to its anti-inflammatory impact (Gomaraschi et al., 2008) or via an alternative mechanism modulating innate immunity (Zamanian-Daryoush et al., 2013). This is an intriguing property of HDL and rHDL nanoparticles that has also been observed with apo A-I mimetics as well (Su et al., 2012). This feature and other favorable characteristics may eventually allow the use of apo A-I mimetics to replace the full-length protein in rHDL nanoparticle formulations.

## Reconstituted Lipoproteins and rHDL vs Liposomes

Because liposomes are the most widely used drug delivery vehicles, especially for cancer chemotherapy, it is worthwhile to compare their performance with lipoprotein based drug delivery vehicles in general and with rHDL in particular. Liposomes are spherical lipid based bilayers, generally with a diameter in the 100–200 nm range, with the capacity to improve the delivery and therapeutic efficacy of intravenous drug formulations (Bozzuto and Molinari, 2015). Lipoproteins, especially HDL, appear to have two major advantages over liposomes as drug delivery agents. First, lipoproteins, including, synthetic lipoprotein formulations, tend to be much smaller (10–50 nm diameter) than liposomes. These feature maybe a significant advantage as the drug carrying nanoparticles are designed with the aim of effectively penetrating the tumor environment. Secondly, both LDL and HDL interact with cell surface receptors via their surface components and thus may have the capacity to reach selective cellular targets depending on the expression levels of specific receptors (see next section). Liposomes, on the other hand, need to be conjugated with antibodies or other targeting moieties that in all likelihood complicate the assembly process and also results in extended costs of preparation.

## Receptor Mediated Drug Delivery via HDL Type Nanoparticles

The natural payload of circulating HDL particles, cholesteryl esters, are known to be primarily delivered via a specific mechanism (selective delivery) using the scavenger receptor type B1 (SR-B1) receptor (Steinberg, 1996; Connelly and Williams, 2004). This mechanism has been linked to a fortuitous finding of SR-B1 overexpression in cancer cells when compared to normal cells (Lacko et al., 2002). Since then several other studies have confirmed that most malignant cells and tumors overexpress this receptor (Zhang et al., 2009; Mooberry et al., 2010; Shahzad et al., 2011; Yang et al., 2013; Ding et al., 2014), perhaps to meet their need for cholesterol, demanded by their high rate of proliferation (Cruz et al., 2013). These findings led us to propose an rHDL drug delivery hypothesis (Lacko et al., 2006) that allows the selective delivery of anti-cancer agents to cancer cells and tumors via the SR-B1 receptor thus unloading of the drug payload from rHDL nanoparticles to cancer cells. This mechanism could allow a “Trojan Horse” strategy (Lacko et al., 2006) that may be particularly effective against cancer cells and tumors.

## HDL Type Nanoparticles as Drug Carriers for Cancer Chemotherapy

Reconstituted HDL type nanoparticles have been extensively studied as drug delivery agents for the purpose of developing enhanced chemotherapy strategies (McConathy et al., 2008; Zhang et al., 2010; Shahzad et al., 2011; Ditiatkovski et al., 2013; Yang et al., 2013). Combined with the receptor mediated drug delivery concept, rHDL nanoparticles may have broad applicability in therapeutics, especially for cancer therapy because during pre-clinical studies, normal cells, for the most part, were found to be shielded from the toxic effect of drugs, opening the way for designing novel chemotherapy regimens via rHDL with much reduced side effect (Sabnis et al., 2012, 2013).

The overexpression of the SR-B1 receptor by most cancer cells and tumors (Lacko et al., 2002; Zhang et al., 2009; Mooberry et al., 2010; Shahzad et al., 2011; Yang et al., 2013; Ding et al., 2014) appears to be a key feature of malignant transformation and development (Cruz et al., 2013). It is a nearly universal feature of malignant cells and tissues, a biological group that is known for its extreme heterogeneity. Consequently, the overexpression of the SR-B1 receptor by malignant cells and tissues may be of significant value in cancer therapeutics.

1. As mentioned above, tumor specific delivery of anti-cancer agents may be possible while protecting normal tissues and thus avoiding side effects.
2. This approach may also be applicable to personalized (or precision) therapy where tumor biopsy samples may be assessed for their SR-B1 expression to select those patients that are most likely to be responsive to therapy.
3. The tumor selective drug delivery concept may have particular relevance to pediatric cancer therapy (Sabnis et al., 2015a; Sabnis et al., 2015) where the immediate and delayed side effects of drug treatment induce particularly severe consequences.

## HDL as a Drug Carrier for Treating Cardiovascular Disease

There have been only a handful of reports describing the potential use of rHDL nanoparticles for the treatment of cardiovascular disease. These include Tanshinone IIA loaded reconstituted high density lipoproteins (TA-rHDL) for atherosclerotic plaque targeting mechanism in a foam cell model (Zhang et al., 2012). The pharmacokinetic studies in rabbits have shown that TA-rHDL was a long-circulating, safe and potentially targeted carrier for delivering lipophilic cardiovascular drugs (Vickers et al., 2011). They also showed that cholesterol homeostasis is coordinated by microRNA 223 via post transcriptional control of multiple genes in lipoprotein and cholesterol metabolism.

Vickers et al., 2011 showed that HDL transports endogenous miRNAs and delivers them to recipient cells with functional targeting capabilities. rHDL injected into mice retrieved distinct miRNA profiles from normal and atherogenic models (Vickers et al., 2011). HDL delivery of both exogenous and endogenous miRNAs resulted in the direct targeting of messenger RNA reporters. HDL-miRNA from atherosclerotic subjects induced differential gene expression, with significant loss of conserved mRNA targets in cultured hepatocytes. The potential for rHDL in cardiovascular therapeutics via the payload being delivered to the endothelial surface is a rapidly growing and potentially effective area for treating atherosclerosis and related abnormalities.

## Nucleic Acid Delivery via HDL Type Nanoparticles

Nucleic acids, including anti-sense nucleotides, siRNA and non-coding RNAs have been used extensively to modulate gene expression and more recently have been considered for therapeutic applications (Bali and Kuner, 2014; Farooqi et al., 2014; Kaur et al., 2014). Although these nucleic acids may be utilized as therapeutic agents, certain major issues limit their utility for clinical applications. These include their sensitivity toward serum nucleases which markedly reduces their serum half-life (Yin et al., 2014), their anionic surface charges that prevent them from penetrating the cell membrane, and their immune-stimulatory potential that triggers the immune response (Dobrovolskaia and McNeil, 2015). Additionally, low therapeutic efficiency of nucleic acids has also been reported due to their non-specific bio-distribution and subsequent off target effects (Zhu and Mahato, 2010).

Subsequently, extensive research on several delivery strategies has led to the design of novel transport systems that are likely to overcome most, if not all, of these barriers to effective nucleic acid therapeutics (Lovell et al., 2011; Shahzad et al., 2011; Yang et al., 2011; Jin et al., 2012; Ding et al., 2014; Pensado et al., 2014; Pandey and Kanduri, 2015). Earlier, Bijsterbosch et al. (2000) showed that cholesterol-nucleic acid conjugates facilitated the accumulation of phosphorothioate oligo-deoxynucleotides (ODN) in liver cells to achieve anti-sense therapy of liver disease. Wolfrum et al. (2007) showed that after systemic administration the lipid-conjugated siRNAs are capable of inducing the silencing of specific genes in the liver.

Vickers et al. (2011) showed that HDL transports endogenous miRNAs and delivers them to recipient cells with functional targeting capabilities. Furthermore, rHDL injected into mice was observed to retrieve distinct miRNA profiles from normal and atherogenic models. HDL delivery of both exogenous and endogenous miRNAs resulted in the targeting of specific messenger RNA reporters. HDL-miRNA complexes from atherosclerotic subjects induced differential gene expression, with significant loss of conserved mRNA targets in cultured hepatocytes. Overall, these observations indicate that HDL participates in a mechanism of intercellular communication involving the transport and delivery of miRNAs to and from cells that express SR-B1 receptor (Vickers et al., 2011). Furthermore, these findings suggest that rHDL is likely to be an efficient carrier for the targeted delivery of miRNA and other low molecular weight polynucleotides for therapeutic purposes.

Accordingly, the delivery of siRNA via lipoprotein carriers has been studied extensively (Lovell et al., 2011; Shahzad et al., 2011; Yang et al., 2011; Jin et al., 2012; Ding et al., 2014) while considerably less information is available on miRNA delivery (Vickers and Remaley, 2012).

An HDL-mimicking peptide-phospholipid nanoscaffold (HPPS) for siRNA delivery has been developed by Yang et al. (2011) with the unique capability of cytosolic delivery of cholesterol-conjugated siRNA. The mechanism of siRNA delivery via these nanoparticles involves the bypass of endolysosomal trafficking and selective delivery of the RNAi to targeted subcellular compartments. In another study, McMahon et al., 2015 described a gold nanoparticle-templated HDL platform (HDL AuNP) for gene therapy. This strategy combines lipid-based nucleic acid delivery that mimics the function of HDL (Vickers et al., 2011). These HDL AuNPs have also been shown to adsorb anti-sense cholesterol-conjugated DNA with subsequent internalization of these conjugates by human cells.

## Conclusion

The concept of delivering therapeutic agents via HDL type transport vehicles has come a long way since the original postulates of Counsell and Pohland (1982) as the receptor mediated uptake of drugs and the extended safe circulation time are attractive features of these nanoparticles. The rHDL drug delivery model may have particularly important applications in cancer therapeutics because of the potential for tumor selective delivery of anti-cancer agents (Shahzad et al., 2011). Strengthening the pre-clinical proof of concept will likely pave the way for clinical trials and ultimately the therapeutic application of the rHDL nanoparticles.

### Conflict of Interest Statement

The authors declare that the research was conducted in the absence of any commercial or financial relationships that could be construed as a potential conflict of interest.
